# The pedunculopontine tegmental nucleus—A functional hypothesis from the comparative literature

**DOI:** 10.1002/mds.26556

**Published:** 2016-02-16

**Authors:** Nadine K. Gut, Philip Winn

**Affiliations:** ^1^ BiozentrumUniversity of BaselBaselSwitzerland; ^2^ Strathclyde Institute of Pharmacy & Biomedical SciencesUniversity of StrathclydeGlasgowUnited Kingdom

**Keywords:** Acetylcholine, Basal ganglia, Cognition, Dopamine, Freezing of gait

## Abstract

We present data from animal studies showing that the pedunculopontine tegmental nucleus—conserved through evolution, compartmentalized, and with a complex pattern of inputs and outputs—has functions that involve formation and updates of action–outcome associations, attention, and rapid decision making. This is in contrast to previous hypotheses about pedunculopontine function, which has served as a basis for clinical interest in the pedunculopontine in movement disorders. Current animal literature points to it being neither a specifically motor structure nor a master switch for sleep regulation. The pedunculopontine is connected to basal ganglia circuitry but also has primary sensory input across modalities and descending connections to pontomedullary, cerebellar, and spinal motor and autonomic control systems. Functional and anatomical studies in animals suggest strongly that, in addition to the pedunculopontine being an input and output station for the basal ganglia and key regulator of thalamic (and consequently cortical) activity, an additional major function is participation in the generation of actions on the basis of a first‐pass analysis of incoming sensory data. Such a function—rapid decision making—has very high adaptive value for any vertebrate. We argue that in developing clinical strategies for treating basal ganglia disorders, it is necessary to take an account of the normal functions of the pedunculopontine. We believe that it is possible to use our hypothesis to explain why pedunculopontine deep brain stimulation used clinically has had variable outcomes in the treatment of parkinsonism motor symptoms and effects on cognitive processing. © 2016 International Parkinson and Movement Disorder Society

The pedunculopontine tegmental nucleus (which will be referred to as the pedunculopontine) has been the focus of much clinical interest in the past few years, notably with regard to the possibility that it could be a target for deep brain stimulation in parkinsonism and related disorders. This interest is predicated on particular views about the functions of the pedunculopontine, largely bound up in the idea that it is a motor structure. In this review, we present data from animal studies showing that the pedunculopontine—conserved through evolution, compartmentalized, and with a complex pattern of inputs and outputs—has functions that go considerably beyond this. It does have motor functions, but as with the basal ganglia (to which it is intimately connected), these appear to do with the formation and updating of action–outcome associations and decision making rather than just the control of coordinated stepping. Moreover, the pedunculopontine has sensory and attentional functions that enable it to take part in making very rapid action selection when needed.

## Anatomical Connectivity and Species Differences

**Figure 1:**
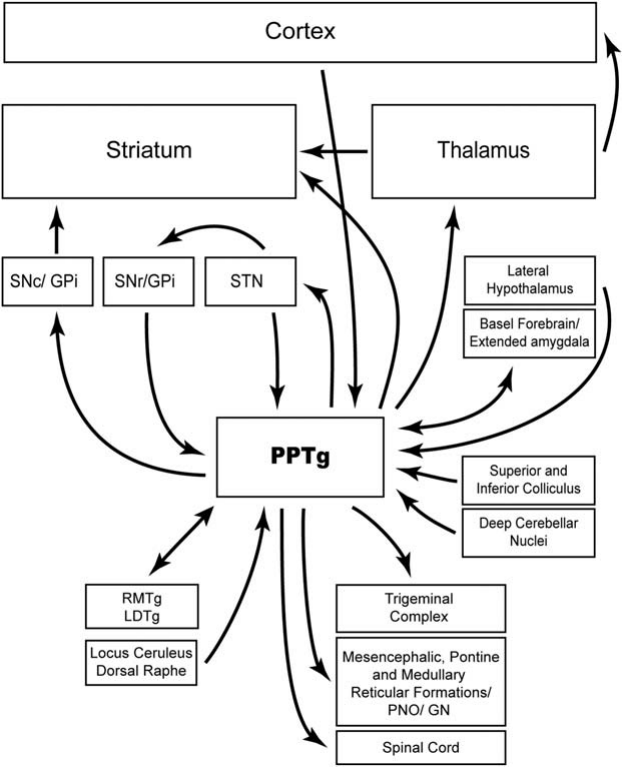
Schematic illustration of pedunculopontine connectivity. Distinct functional types of pedunculopontine subpopulations innervate basal ganglia and in turn basal ganglia structures project back to different neuronal populations in the pedunculopontine. It is important to note that projections form the pedunculopontine to the structures illustrated here are not wholly independent: cholinergic and noncholinergic neurons from topographically distributed populations send collaterals to several structures (eg, to thalamus and basal ganglia). Likewise, descending collaterals of ascending axons contribute to a dense innervation of structures in the lower brainstem, pons, medulla, and spinal cord.^125^

Comparative studies of the anatomy of the pedunculopontine show that it has a broadly similar construction and pattern of connections in all vertebrate species: (i) internal segregation into a *pars dissipatus* and *pars compactus* (corresponding to the terms anterior and posterior pedunculopontine used by our lab in rodent studies) (The *pars dissipatus* is the bulk of what in the past has been called the midbrain extrapyramidal area,^1^ reflecting the fact that it receives considerable output from the basal ganglia.); (ii) a significant population of large cholinergic neurons interdigitated with smaller noncholinergic neurons (containing primarily gamma aminobutyric acid [GABA] and glutamate) distributed differentially through anterior/posterior and mediolateral gradients; (iii) extensive connections with the brain stem (both motor and autonomic systems), spinal cord, and cerebellum; (iv) sensory input from visual, auditory, and tactile systems as well as elements of the ascending reticular activating system; (v) significant inputs to the thalamus, giving rise to the ability to effect cortical activity; and (vi) reciprocal connections with the basal ganglia and associated limbic structures (see Table 1 and Fig. 1).

**Table 1:** Principal connections of the pedunculopontine tegmental nucleus; representative references are given for each cluster

Midbrain, brain stem, cerebellum, and spinal cord	
Inferior and superior colliculus (reciprocal)	96, 97, 98
Pontine and medial reticular formation; nucleus pontis oralis	99, 100, 101, 102
Motor trigeminal	103, 104, 105
Medulla	99, 106
Spinal cord (reciprocal)	107, 108, 109
Ascending reticular activating system	
Dorsal raphe, locus coeruleus, laterodorsal tegmental nucleus	70, 107, 110, 111
Forebrain	
Thalamus	112, 113, 114, 125
Basal ganglia—striatum; globus pallidus (internal and external); subthalamic nucleus; substantia nigra pars reticulata; and projections to midbrain dopamine‐containing neurons	47, 107, 115, 116, 117, 118, 119, 120
Extended amygdala, basal forebrain, lateral hypothalamus	113, 118, 121, 122

Cortical influence is mainly via connections through the thalamus. There is some evidence for direct projections to medial and sulcal frontal cortical areas.^117^ Auditory^123^ and motor^124^ cortex send projections to pedunculopontine tegmental nucleus.

The connections of the pontomedullary tegmentum in a variety of species were first investigated by Elizabeth Crosby and her colleagues in the 1930s. Those studies showed strong cross‐species similarities in connectivity, which were confirmed in later experiments. It has been shown in various species that the pedunculopontine is highly conserved through evolution, with a similar structure and pattern of connections in teleost fish, amphibians, birds, mammals, and primates (including human).^1^, ^2^, ^3^, ^4^, ^5^, ^6^, ^7^, ^8^ Anatomically, the differences between species are a matter of degrees rather than being fundamentally different. For example, the relative strength of afferent and efferent connections of pedunculopontine with the substantia nigra pars reticulata and medal segment of the globus pallidus (which in rodents is the entopeduncular nucleus) differ between species.^9^ It has been argued that evolution from quadrupedal to bipedal gait has been a cause of differences between species.^9^ Significant species differentiation might be suggested by the observation of one significant disparity in outcome after pedunculopontine lesions in primates when compared with all other species. In nonprimate species, repeated studies have shown that there are no gross motor deficits after a bilateral loss of the pedunculopontine,^10^, ^11^, ^12^, ^13^, ^14^, ^15^, ^16^, ^17^, ^18^ but akinesia or gait disturbances appear in primates after lesions.^19^, ^20^, ^21^, ^22^ However, important points in methodology are as (or more) likely to be the cause of this single point of differentiation as is anatomical structure. In particular, the lesions producing frank motor impairments in primates were made using radiofrequency ablation (which destroys fibers as well as neurons and glia) or kainate, a highly potent neurotoxin needing concentration orders of magnitude lower than those used with other excitotoxins and that typically spreads well beyond the intended target. Comparable doses in rats produce very large lesions, which is why kainate stopped being used as a toxin of choice for making excitotoxic lesions in rodent studies more than 20 years ago.^23^ It is also important to note that electrophysiological studies of single unit activity in primate pedunculopontine have shown patterns of activity consistent with interpretations of pedunculopontine function that are based on data from rodent studies, indicating integrative rather than purely motor functions for the pedunculopontine.^24^

## The Functions of the Pedunculopontine

The animal literature has seen considerable changes in thinking regarding the pedunculopontine during the past decade. The extent of its connectivity and structural heterogeneity underpins the fact that it is involved in diverse processes: autonomic functions, movement and sensorimotor coordination, sleep–wake regulation, attention, and learning. The traditional account of pedunculopontine as part of the ascending reticular activation system involved with sleep regulation^25^ remains viable. However, firing pattern changes in pedunculopontine that relate to different brain states are complex and do not reflect a role for the pedunculopontine as a “master switch” for behavioral state control.^26^ Many brain structures have a role in sleep regulation^27^ with no single structure in overall control. Neurotoxic destruction of the pedunculopontine leaves sleep patterning intact, but changes the ability of animals to respond to challenges such as deprivation of rapid eye movement sleep.^25^ Recent studies^28^ have demonstrated that different populations of neurons in the pedunculopontine—cholinergic and noncholinergic—have different roles in behavioral state control. During cortical slow wave states, pedunculopontine neurons are synchronized locally and to cortical oscillatory activity, but during activated states noncholinergic neurons show tonic discharge with little responsiveness to transitions across states. Cholinergic neurons show phasic short latency responses to sensory stimulation—their responding in the activated state is uncoordinated and does not appear to be involved in the maintenance of wakefulness.^28^ Rather, these neurons appear to be involved in the processing of sensory information. This involvement in the control of thalamocortical activity in the activated (waking) state gives the pedunculopontine very significant potential in regulating cortical processing across a variety of domains. Functional^29^ and anatomical studies demonstrate pedunculopontine control over thalamic nuclei, including reticular and intralaminar nuclei (see Table 1). Connections through the thalamus with thalamic nuclei give access, for example, to diverse cortical regions, including prefrontal and motor cortex, cingulate, and entorhinal cortices.

The other traditional view of pedunculopontine function—that it is critical for locomotion as part of the so‐called mesencephalic locomotor region (a functionally rather than anatomically defined area)—appears to be no longer tenable based on the bulk of animal studies. The association of the pedunculopontine (and the immediately adjacent cuneiform nucleus) with locomotion came from experiments involving electrical stimulation in the area of the pedunculopontine that elicited coordinated locomotion, sometimes described as machine like. An examination of the mesencephalic locomotor region was typically done in mesencephalic preparations (ie, animals in which descending control of the pedunculopontine and cuneiform nucleus had been severed).^30^ However, serial studies in rat, mouse, and cat have shown that pedunculopontine loss does not impair movement per se as tested in photocell cages, the home cage, circular corridor, or in open fields^10^, ^11^, ^12^, ^13^, ^14^, ^15^, ^16^, ^17^ (and it is worth noting that excitotoxic lesions of the cuneiform nucleus do not impair locomotion either).^31^ Likewise, pedunculopontine loss does not affect drug‐induced locomotion.^11^, ^12^, ^13^, ^14^, ^15^ At a more subtle level of function, our recent data shows that neither full nor partial pedunculopontine lesions affect gait parameters such as stride length, base of support, and swing speed.^18^ However, performance does decline when excitotoxic lesioned rats are faced with tasks that demand forced acceleration, and grasping tasks are similarly affected.^32^ Overall, recent literature suggests that there is no gross motor dysfunction after pedunculopontine destruction, but that subtle motor deficits are present and related to task demand. Why should electrical stimulation have such potent effects? A key to understanding this is the fact that the clearest demonstrations of a mesencephalic locomotor region came from local stimulation in transected animals where descending control of the pedunculopontine and cuneiform nucleus had been lost. The descending fibers from the basal ganglia primarily contain the neurotransmitter GABA and work to inhibit activity; that is, it stops the pedunculopontine from taking control of descending motor output (from the pontine and medullary reticular formations). Losing inhibitory control and then stimulating inevitably drives descending pedunculopontine/cuneiform nucleus output and produces locomotion. Similarly, we have seen in unpublished studies that local inhibition of GABA activity in the pedunculopontine produces explosive motor behavior. The functional effects that led to the idea of a mesencephalic locomotor region are perfectly sound and have been easily replicable. What is at issue is interpretation. The data do not imply a purely locomotor role for the pedunculopontine.

An increasing amount of animal literature indicates that the pedunculopontine has a role in cognitive functions, that is, learning and reinforcement processes, the updating of action–outcome associations, and decision making.^17^, ^24^, ^32^, ^33^, ^34^, ^35^, ^36^, ^37^, ^38^, ^39^, ^40^ Deficits in cognitive performance following lesions cannot simply be ascribed to poor motor performance. For example, on the 8‐arm radial maze, lesioned rats are quicker than controls moving from arm to arm. What is defective is their decision making about which arm to enter.^37^ Again, task demand might be an important consideration. When required only to associate one of two places with reward (sucrose‐motivated conditioned place preference), pedunculopontine lesioned rats perform successfully^41^ but behave at chance levels in the more complex 8‐arm radial maze.^37^, ^42^ What appears to be impaired is the ability to form associations. An example of this is the work of Alderson and colleagues, who showed that pedunculopontine lesioned rats could not learn to lever press for drug reward unless they had been pretrained. If they had learned lever press–reward association prior to surgery, their subsequent performance was unimpaired.^33^ Lesioned rats can do the task, but cannot learn it. That this represents action–outcome association specifically was confirmed later by MacLaren and colleagues^43^ using a contingency degradation test in which rats with the pedunculopontine inactivated by local injection of muscimol failed to respond properly to changed contingencies in a learning task. They did not stop responding (as the controls did) when action–outcome contingencies shifted.

## Differentiation in the Pedunculopontine: Which Neurons Do What?

The pedunculopontine contains a population of large cholinergic neurons (Mesulam's Ch5 group^44^) as well as GABA and glutamate neurons in addition to peptidergic neurons containing, for example, substance P or atrial natriuretic peptide.^44^, ^46^ Cholinergic neurons also possess nicotinamide adenine dinucleotide phosphate diaphorase (the synthetic enzyme for nitric oxide) and have been seen to coexpress glutamate, GABA, and substance P.^47^, ^48^ The posterior part of the pedunculopontine—the pars compactus—contains most (although not all) of the cholinergic neurons. Analogies with the structure of substantia nigra have previously been drawn 49—in both cases there is a compact portion containing either cholinergic or dopaminergic neurons with long and widespread projections and a less cell dense portion containing other neurons with more restricted projections and regulated by basal ganglia outflow. Although the size and electrophysiological activity of cholinergic neurons clearly differentiate them from noncholinergic neurons—and although the actions of acetylcholine in target structures such as the thalamus, substantia nigra/ventral tegmental area, and medulla have been described—until recently there has been no opportunity to examine the behavioral functions of these cholinergic neurons selectively. The development of a fusion toxin (urotensin II/diphtheria toxin)^50^ made examinations of the specific functions of these neurons possible. Excitotoxic lesions of the pedunculopontine destroy all local neurons (and not fibers of passage) and have been seen to impact reward‐related responding and learning, as noted previously. Comprehensive and selective lesions of cholinergic neurons do not impact responding and learning. There is no effect on reward processing,^32^, ^51^ locomotion, or learning.^52^ Reported gait deficits in primates after urotensin II/diphtheria toxin lesions were compromised because the movement problems only became apparent with doses of urotensin II/diphtheria toxin that produced nonspecific lesions.^53^ Unlike the effects of excitotoxic lesions, skilled motor performance is similarly unaffected by urotensin II/diphtheria toxin lesions. Rats handle small edible objects without difficulty, but deficits emerge when pedunculopontine urotensin II/diphtheria toxin lesioned rats are challenged to maintain stability on an accelerating rotarod treadmill.^32^ However, whether this is a motor deficit or an attentional problem is uncertain. In rodents, excitotoxic^54^ as well as selective urotensin II/diphtheria toxin pedunculopontine cholinergic lesions lead to deficits in sustained attention^55^ and acoustic startle responding. A selective loss of pedunculopontine cholinergic neurons reduced this to the point where it was not detectable, but when intertrial intervals were long enough appropriate responding was seen.^56^ This returns the cholinergic neurons to a more prominent ascending reticular activing system function, maintaining vigilance and alertness (compatible with a role in behavioral state control) leaving the noncholinergic neurons apparently with greater responsibility for the more basal ganglia–like problems of action selection and learning (although these of course will also benefit from appropriately targeted attention mediated by pedunculopontine cholinergic neurons). Further dissection of the behavioral functions of particular neuronal populations remains a high priority for understanding pedunculopontine functions.

## A Functional Hypothesis

The control of action by the CNS is a complex process involving an analysis of sensory data, comparison with past experience, and a selection of one among many competing potential actions—and the need to refresh, switch, or stop the selected action when appropriate. The basal ganglia are thought to provide a central selection mechanism for choosing competing alternative actions.^57^ The pedunculopontine can be understood as a part of this action selection process but at a lower level of the neuraxis, providing input to thalamo‐cortico‐striatal circuity (through connections with midbrain dopamine [DA] neurons, elements of the basal ganglia, and the thalamus) and receiving output from the basal ganglia. The bulk of this basal ganglia input is inhibitory (the exception being glutamatergic input from the subthalamic nucleus) and appears to control efferents descending from the anterior pedunculopontine, preventing impulsive responding to sensory input in circumstances when it would be inappropriate to do so. This inhibitory basal ganglia output to pedunculopontine is evidently involved in the production of parkinsonian symptomatology. Evidence for this inhibition comes from, for example, the fact that metabolic mapping using 2‐deoxyglucose in primates which had been made hemiparkinsonian by 1‐methyl‐4‐phenyl‐1,2,3,6‐tetrahydropyridine (MPTP) injection highlighted overactivity in the pedunculopontine.^58^ In addition stimulation or local injections of the GABA antagonist bicucculine attenuated akinesia in primates similarly made parkinsonian with MPTP.^59^, ^60^ Like others, we are confident that the pedunculopontine is involved in movement, but we stress that the nature of this involvement is not simply with the control of coordinated locomotor activity but with action selection. The pedunculopontine is in a position to make immediate decisions when there is an imperative need to do so and at moments when comprehensive processing through forebrain circuitry would be too slow. Critical to this is the availability of short latency sensory data, descending connections to sites of motor and autonomic control, and prevention of impulsive responding by inhibitory outflow from the basal ganglia.

**Figure 2:**
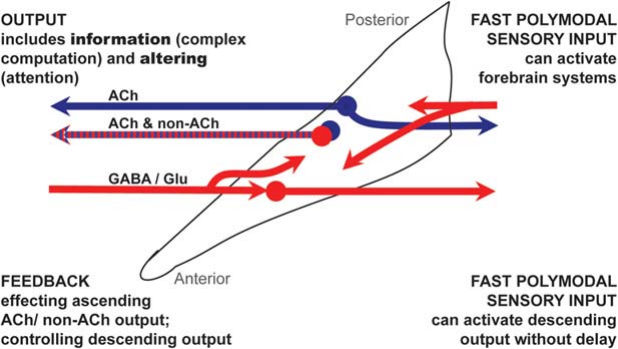
Schematic illustrating the functional inputs and outputs of the pedunculopontine. The dotted line outline of pedunculopontine is taken from the atlas of Paxinos and Watson.^95^ The colored representation of cholinergic (blue) and noncholinergic neurons (red) is illustrative only; discussion of the topological organization of these can be found elsewhere.^7^, ^126^ Sensory input arrives predominantly in the posterior pedunculopontine (pars compactus) and influences ascending activity (which delivers information and guides attention) and has the capacity to stimulate immediate responses to stimuli, likely in advance of any forebrain processing (the acoustic startle response for example^56^. Input from the forebrain (much of it from the basal ganglia) is largely (but not exclusively) inhibitory, regulating response generation from within the pedunculopontine. It is worth noting that in 1955, building on the analysis of the ascending reticular activating system made by Moruzzi and Magoun^127^ as well as psychological drive theories, Hebb^128^ observed that “in general terms, psychologically, we can now distinguish two quite different effects of a sensory event. One is the cue function, guiding behavior; the other, less obvious but no less important, is the arousal or vigilance function. Without a foundation of arousal, the cue function cannot exist” (p. 249). What we are proposing here is highly reminiscent of this—that the pedunculopontine has the capability to (i) deliver information to forebrain systems^24^, ^64^, ^65^, ^70^; (ii) change the electrophysiological activity of thalamocortical and brainstem circuitry^26^, ^28^, ^102^, ^125^; (iii) maintain attention^54^, ^55^; and (iv) initiate rapid responding when required, further developing on Hebb's idea.^56^, ^72^

To unpack this idea, we first note that the function of the pedunculopontine must reflect the fact that it is differentiated internally (see Fig. 2). Short latency sensory data appears to be received in the posterior part of the pedunculopontine (pars compactus). Electrophysiological recordings in cats and rats show that the neurons have exceptionally short activation latencies to sensory stimuli,^61^, ^62^, ^63^ and in primates pedunculopontine neurons analyze sensory information regarding the salient aspects of a stimulus, that is, the association of a stimulus with a reward, the prediction of the reward value, the reward itself, and the actual value of the reward.^24^, ^64^, ^65^ Similar data have been shown in mice,^40^ indicating commonality of function across species. Kobayashi and colleagues^24^, ^64^, ^65^ have shown repeatedly that patterns and rates of pedunculopontine neuron firing in primates are not just related to movements (visual saccades) and that the subjects—macaques—had to perform a reward task. Different populations of pedunculopontine neurons responded to reward‐predicting stimuli and their delivery, mirroring the expected reward value.^24^, ^64^, ^65^ Tonic increases and decreases during task execution correlated with response magnitude to larger or smaller reward cues.^64^ Likewise, when the mice had to make a left‐ or right‐orientating movement following an odor cue to receive a reward, pedunculopontine activity was related to direction selection. Overlapping populations were firing in relation to movement direction and reward outcome.^40^ Pedunculopontine neurons have an ability to pass information on to basal ganglia systems involved in the considered selection of one among many possible actions. For example, they do this by providing midbrain DA neurons with information about incoming sensory stimuli. This happens fast enough (pedunculopontine sensory response latencies are between 4–80 milliseconds^61^, ^62^ to lead us to assume that the pedunculopontine informs DA neurons (which have a response latency of 70–100 milliseconds after stimulus presentation) about external sensory events.^66^ By influencing midbrain DA neurons^67^ and activity in the thalamus, the pedunculopontine can assume a role in the construction of goal‐directed actions and movements. It has the capacity to deliver fast sensory data with value already partly assessed to effect corticostriatal processing. In parallel, it is likely that pedunculopontine cholinergic neurons have a simultaneous role in focusing attention and initiating a rapid response to imperative signals.^55^, ^56^ The pedunculopontine clearly directs output into forebrain systems, but in addition it projects to motor and associated autonomic control sites lower in the brain stem,^8^, ^68^, ^69^ allowing regulation of key motor, respiratory, and cardiovascular sites that are involved in the production of rapid responses to imperative stimuli. (See Table 1 for details of pedunculopontine connections with the pontine reticular formation, medulla, and spinal cord.) This possession of ascending and descending output is what gives the pedunculopontine the ability to engage in action selection processes through thalamo‐cortico‐striatal systems while influencing behavior more immediately through direct descending connections.

The pedunculopontine is a deep part of basal ganglia outflow circuitry. Corticostriatal outflow predominantly arrives in the anterior pedunculopontine. Much of it comes from the basal ganglia output nuclei (substantia nigra pars reticulata and the internal segment of the globus pallidus) and is GABA mediated (the exception being glutamatergic input from the subthalamic nucleus), which holds descending pedunculopontine outflow in check. It receives processed information from basal ganglia output nuclei, relating it and comparing it to immediate ongoing sensory input before the information either continues to leave the basal ganglia, is blocked, or reentered to the corticostriatal system for further processing. Behavioral findings support this distinction. Lesions of posterior pedunculopontine impair instrumental learning, presumably interfering with the necessary input to midbrain DA neurons. Anterior pedunculopontine lesions have no effect on learning rate, but cause deficits that can be described as behavioral disinhibition or disorganization, likely the result of a disturbance of basal ganglia outflow.^17^, ^69^, ^70^

Therefore, a distinction is made between the anterior pedunculopontine (a basal ganglia output station) and the posterior pedunculopontine, which makes a first pass analysis of sensory data and, if required, initiates a rapid response before the data are fully processed by forebrain systems. The short response latency to stimuli, involvement in learning, and its role in startle responses and prepulse inhibition^36^, ^56^, ^71^, ^72^ support the idea that the pedunculopontine has the capacity to act on the brain stem without involving the basal ganglia and that it is involved in learning when not to respond.^43^ It is imperative that animals have the capacity to make such immediate judgments about the need to act in particular situations (as well as being able to make more deliberate and considered decisions when time permits). Critically, there must be a mechanism to prevent such a system from making impulsive responses: it must be appropriately braked, which is the pedunculopontine. The pedunculopontine is not the only structure involved in this. Similar arguments have been made for the superior colliculus. What perhaps makes the pedunculopontine unique is its capacity for assessing the motivational value of inputs, which necessarily has to involve learning, its polymodal sensory input, and its ability simultaneously to effect lower brain action systems (such as the pontine reticular formation) as well as thalamo‐cortico‐striatal processing.

## What Does This Mean for the Neurological Literature in Humans?

Clinical trials of deep brain stimulation (DBS) in the pedunculopontine show heterogeneous results and an overall lack of consistent improvement. The first studies were encouraging.^73^, ^74^, ^75^ The authors reported safe surgical implants and subjective reports from patients of an improved feeling of well‐being and improved motor symptoms. Subsequent studies did not deliver the same results and early improvements could possibly be attributed to placebo effects—follow‐up studies reported only transient gait amelioration.^76^ The first double‐blind trials proved the importance of careful assessment of stimulation. These studies did not show motor improvements with pedunculopontine‐DBS as measured by the UPDRS^77^, ^78^, ^79^ or by objective freezing measures.^77^ The freezing of gait and/or falls were reported to improve with pedunculopontine‐DBS, subjectively^77^ and objectively,^80^ but not gait parameters, such as stride length. StartReact—an acceleration of a response after a loud auditory stimulus—was shown to be deficient in patients with freezing problems and could be restored by pedunculopontine‐DBS.^81^ Reaction time in various attentional^82^ and working memory tasks^83^, ^84^ improved, but not the accuracy of responses; improvement was also found in verbal fluency, executive functioning, and delayed recall.^83^, ^85^ Furthermore, it was reported that an increase in the duration of rapid eye movement sleep could be achieved with pedunculopontine‐DBS.^86^, ^87^ Improvements in attention, memory, and executive function are all consistent with what has been described in experimental studies of nonprimate species.

The pedunculopontine is undoubtedly involved in Parkinson's disease through a loss of neurons and an altered state consequent upon basal ganglia dysfunction—excessive GABA‐mediated inhibition of the anterior parts of the pedunculopontine. What the animal literature predicts—and has been picked up by the literature on humans^88^—is that the effects of interference with the pedunculopontine through DBS will be critically dependent on the location of the stimulation and its parameters. Specific targeting within pedunculopontine of parkinsonian patients has varied. An Italian group implanted electrodes in caudal pedunculopontine,^89^, ^90^ but others were placed rather rostrally^83^, ^91^ (if the pedunculopontine was targeted at all^92^). Prompting the activity of pedunculopontine neurons themselves or their inputs will have profoundly different effects. Stimulation in the posterior part will affect thalamocortical and thalamostriatal processing. In a parkinsonian rodent model, the relation between cortical and pedunculopontine activity was shown to be altered,^93^ but given that the essential pathology of the basal ganglia will not have been affected by the DBS, this might be no more than a marginal benefit. The same stimulation in the anterior part of the pedunculopontine we would expect, on the basis of animal studies, to be more difficult. The need here is both to eliminate the chronic dysfunction produced by changed basal ganglia input and to restore a normal level of braking of pedunculopontine activity that can be released on demand. It would be preferable to have an action on the inputs to the anterior pedunculopontine rather than the neurons themselves; normalizing the inputs would be better than attempting to activate anterior pedunculopontine neurons themselves while still in receipt of disordered input. We have recently shown that this functional distinction between anterior and posterior pedunculopontine is significant in a rodent model of parkinsonism. We assessed how DBS targeted to either the anterior or posterior pedunculopontine affects gait and postural disturbances in rats bearing extensive loss of striatal DA and in rats with the same DA depletion combined with partial lesions of the pedunculopontine itself, which better mimics the condition that applies in parkinsonism.^18^ Anterior pedunculopontine stimulation increased gait freezing, but posterior stimulation produced mild gait improvement.

## In Summary

Slightly more than 30 years ago David Marsden^94^ wrote about the mysterious motor functions of the basal ganglia. At that time there was considerable debate about whether the basal ganglia had motor or cognitive functions. What the debate reflected was a relatively poor understanding of what is meant by *motor functions*. Likewise, previous thinking about the pedunculopontine has occurred within a framework that regarded brain stem function largely as automatic—certainly not cognitive—and that, for the pedunculopontine, focused on the production of locomotion and sleep. We believe that the new conceptual framework that has been developed is a significant advance on this and that studies of a variety of animal species have helped achieve this because the function of pedunculopontine is a very fundamental one, highly conserved through evolution. As well as being a key part of basal ganglia outflow, the pedunculopontine is a critical mechanism for making swift responses essential for survival, which is evident by its compartmentalized nature, its ability to effect thalamo‐cortico‐striatal processing and be regulated by the basal ganglia, its capacity to assess incoming sensory data, and its connections with motor and autonomic control systems in the lower brain stem and spinal cord. Its demonstrated role in action–outcome association learning, attention, and decision making and the complexity of its information processing at a single unit level are evidence of this. Understanding the pedunculopontine in these terms should enable better clinical appreciation of its value as a target for therapeutic interventions.

## Author Roles

(1) Research Project: A. Conception, B. Organization, C. Execution; (2) Statistical Analysis: A. Design, B. Execution, C. Review and Critique; (3) Manuscript: A. Writing of the First Draft, B. Review and Critique.

N.K.G.: 3A, 3B

P.W.: 3A, 3B

## Full financial disclosures of all authors for the past year, regardless of relationship to current manuscript

This research was supported by Medical Research Council grant G0901332 (PW) as part of a European Research Area Network grant. N.K.G. was also supported by a studentship from the School of Psychology, University of St. Andrews and by an award from a Scottish Universities Life Sciences Alliance Strategic Research Development Grant to the University of Strathclyde. P.W. is currently in receipt of a Biotechnology and Biological Sciences Research Council Strategic Skills award (BB/JO13854/1). Previous support to P.W. from the Wellcome Trust and the Biotechnology and Biological Sciences Research Council is gratefully acknowledged. N.K.G. is currently supported by the Swiss National Science Foundation.
